# Spontaneous rupture of a bicornuate uterus at 19 weeks gestation: A case report

**DOI:** 10.1016/j.ijscr.2025.111239

**Published:** 2025-03-31

**Authors:** Joudi Tarabishi, Fatima Breim, Aya Laila, Hibatullah Alhammoud

**Affiliations:** Department of Obstetrics and Gynecology, University of Aleppo, Faculty of Medicine, Aleppo, Syria

**Keywords:** Bicornuate uterus, Uterine duplication anomalies, Malformation, Uterine rupture, Pregnancy, Case report

## Abstract

**Introduction:**

A bicornuate uterus (BU) is one of the four principal abnormalities resulting from defective embryological development of the Müllerian ducts, associated with increased risks of miscarriage, preterm birth, and malpresentation.

**Case presentation:**

This case report describes a rare instance of spontaneous uterine rupture in a 31-year-old woman with a bicornuate uterus at 19 weeks of gestation. She presented with severe abdominal pain and signs of hypovolemic shock. Ultrasound revealed a single dead fetus, and surgical exploration confirmed uterine rupture with the deceased fetus free in the abdominal cavity and significant hemoperitoneum. The ruptured left uterine horn was excised, and the uterine defect was repaired. The patient's postoperative recovery was uneventful.

**Discussion:**

A bicornuate uterus is a rare anomaly that can lead to serious obstetric complications at any stage of pregnancy. In our case, despite the absence of prior indications of uterine defects, the condition was identified during an emergency diagnostic laparotomy following the exclusion of other surgical diagnoses. Uterine rupture, often linked to previous cesarean deliveries and uterine anomalies, can manifest as acute abdominal pain, vaginal hemorrhage, and fetal distress.

**Conclusion:**

Uterine rupture should be included in the differential diagnosis for acute abdominal pain in mid-trimester pregnancies, particularly in cases of uterine anomalies.

## Highlights

• Uterine rupture must be a differential diagnosis alongside other emergencies in the first and second trimesters.
• A bicornuate uterus raises uterine rupture risk, especially in patients with a cesarean history, increasing incidence.
• Initial management involves surgical resection of the ruptured horn after resuscitating the patient’s hemodynamic state.
• Hysterectomy is recommended if the patient chooses to terminate her fertility.

## List of abbreviations

**Table:** 

BU	bicornuate uterus
ED	Emergency Department
BPD	biparietal diameter
FL	femoral length
ICU	intensive care unit
WBC	white blood cells
ABG	arterial blood gases
PROM	premature rupture of membranes
PPROM	preterm premature rupture of membranes

## 1 Introduction

A bicornuate uterus (BU) is a rare congenital anomaly resulting from the incomplete fusion of the two Müllerian ducts during embryonic development, leading to a uterus with two distinct horns. This condition poses significant reproductive challenges and is associated with adverse outcomes [1].

Among the various types of Müllerian duct anomalies, BU accounts for approximately 25 % of cases [2]. Its prevalence varies from 0.4 % in the general population to between 1.1 % and 4.7 % among women experiencing reproductive issues [3].

Women diagnosed with BU may face several obstetric complications, including mid-trimester miscarriages, preterm labor and delivery, malpresentation, and antepartum and postpartum hemorrhage; however, uterine rupture remains a relatively rare event [3].

Rupture of the gravid uterus is a rare but serious obstetric emergency characterized by high morbidity and mortality rates. This complication is more frequently observed in multiparous women or those with a history of uterine surgery, such as cesarean sections or myomectomies, and typically occurs during labor [4]. In contrast, uterine rupture during early gestation—specifically in the first and second trimesters—is uncommon and is often associated with uterine anomalies like a bicornuate uterus or cornual pregnancies [4].

This case report discusses a spontaneous uterine rupture in a woman with a bicornuate uterus during the second trimester of pregnancy, emphasizing the critical need for awareness and careful monitoring in these high-risk situations.

The work has been reported in line with the SCARE criteria [5].

## 2 Case presentation

A 31-year-old G5P4004 woman, who has undergone one cesarean section a year ago, presented to our Emergency Department (ED) in March 2024. The woman was at 19 weeks of gestation according to her family with unmentioned last menstrual period (lactation period). The patient was unconscious with hypovolemic signs (hypotension, thready weak rapid pulse, pale skin color with peripheral cyanosis). Her family mentioned recurrent vomiting episodes with an average of seven times during the past 3 h. At the ED, sudden spasmodic movements occurred lasted for 3 min. The previous four pregnancies were uneventful. There was no history of either curettage or intrauterine device insertion. Additionally, she had no history of drug use, abdominal trauma, or smoking.

On physical examination at the time of admission, she was pale and unconscious, with a tender abdomen, muscle guarding, and unstable vital signs (hypotension = 70/40 mmHg, thready tachycardia = 110 bpm, and temperature = 36.5 °C). A vaginal exam revealed slightly opened external orifice with no observed vaginal bleeding.

Obstetric ultrasound examination showed a single dead fetus (absent fetal cardiac activity), normal amniotic fluid amount, with mostly placenta coverage, biparietal diameter (BPD) = 19 weeks, and femoral length (FL) = 17 weeks.

As there were no apparent signs of an obstetrical issue, the initial management was to enhance the general state of the patient by providing a venous line, maintaining the airway passage open, supplying well-hydration via saline solution and proton-pump inhibitor drugs, inserting a foley catheter, and requesting blood units.

After an extensive pre-operative laboratory tests were conducted (Table 1), three screened, cross-matched whole blood and two fresh frozen plasma units were transfused.

**Table 1:** Laboratory results.

Test	On admission	On discharge	Normal range
Blood group	A+ positive		
Hemoglobin (g/dL)	7.6	8.4	11.0–16.0
Hematocrite (%)	21.4	22.0	36.0–48.0
White blood cells (WBC) (10^3^/μL)	19.7	22.7	4.0–10.0
Platelets (10^3^/μL)	361	206	150–400
Prothrombin time (s)	14.5	14.5	
Activity (%)	77 %	77 %	
INR	1.13	1.13	
C-reactive protein (mg/dL)	30.4		Up to 7
Fasting glucose (mg/dL)	191		
Glucose (mg/dL)	153		
Urea (mg/dL)	33		10–40
Creatinine (mg/dL)	1.64	1	0.6–1.1
Lipase (U/L)	228		Up to 180
Amylase (U/L)	133		Up to 80
Alanine aminotransferase (U/L)	15		Up to 41
Aspartate transaminase (U/L)	22	46	Up to 40
LDH (U/L)	488	623	200–420
Fibrinogen (mg/dL)	440		150–400
Fibrin degradation products (F.D.P) (mg/dL)	7		0–5
D-dimer (ng/dL)	760		0–400
Magnesium (mg/dL)	1.9		1.7–2.4
Total calcium	1.69		
Ionized calcium (mmol/L)	1.23	0.87	1.1–1.3
Sodium (mmol/L)	142	141.9	135–145
Potassium (mmol/L)	3.8	3.98	3.5–5.5
Chloride (mmol/L)	104	106	98–107
Blood film	Red blood cell: normochromic normocytic; white blood cells: leukocytosis; neutrophelia few large immature cells Platelets: normal		

A second abdominal ultrasonography was done after 1 h of admission, it showed excessive free fluid during the peritoneal cavity without organic hematomas. However, chest-X-ray was normal without subdiaphragmatic free gas or visceral perforation signs and an abdominal-X-Ray was normal without occlusion signs.

Two hours later, a general surgical consultation was conducted which revealed no emergency general surgical diagnosis after an extensive physical examination and radiological procedures check.

A diagnostic laparotomy was decided after an informed consent.

**Fig. 1:**
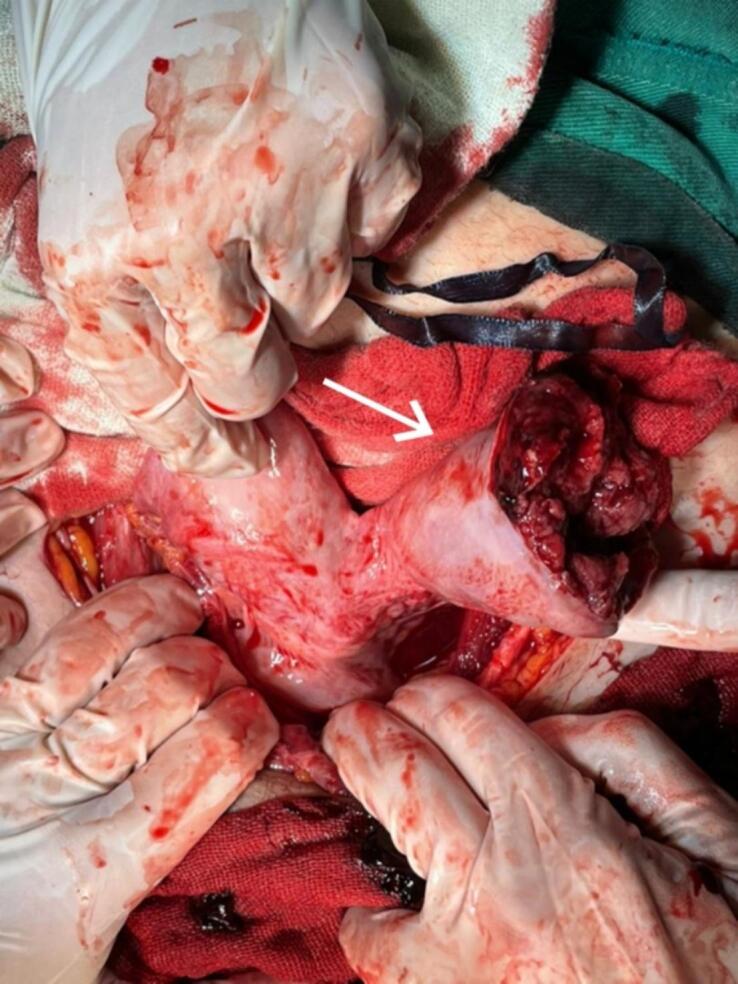
A bicornuate uterus with a ruptured left horn.

**Fig. 2:**
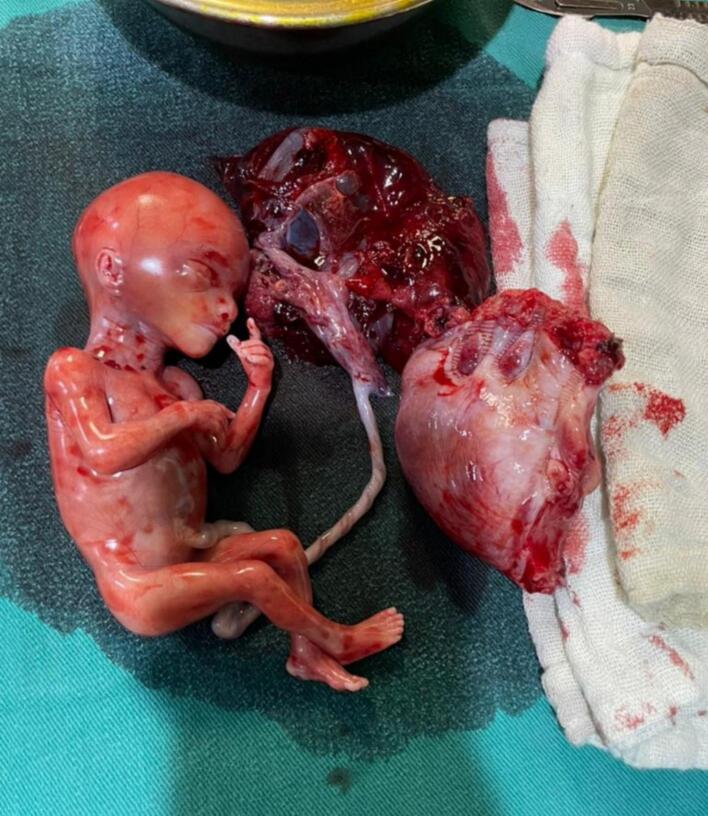
Dead 19-week gestation embryo with attached placenta, and the left resected horn.

Intraoperative findings (Fig. 1) showed: 1) A massive hemoperitoneum of around 1.5 L of blood and blood clots. 2) A bicornuate uterus with a ruptured left horn. 3) Dead 19-week gestation embryo in the abdominal cavity (Fig. 2).

The management included: suctioning the hemoperitoneum, removing the placenta and fetus, and resecting the ruptured uterus horn and forwarding it to the pathology laboratory and suturing the defect using absorbable separated sutures, and eventually inserting two intraabdominal drainages. During the operation, three additional screened, cross-matched whole blood and three fresh plasma units were transfused. The woman was observed at the intensive care unit (ICU) for half a day and then was transferred to the ward where she stayed for one day before discharging.

The woman had an uneventful recovery and was discharged home on the fourth postoperative day. She was counseled about the high risk of recurrence and advised to avoid future pregnancies.

Outpatient follow-up was unavailable as the patient lived in an area with minimal socio-economic situation.

## 3 Discussion

A bicornuate uterus (BU) is a rare type of congenital uterine anomalies. BU is associated with increasing risks of obstetric complications including: miscarriage, placental abruption, premature rupture of membranes (PROM), preterm premature rupture of membranes (PPROM), fetal malpresentation at delivery, preterm delivery, cesarean delivery, fetal and perinatal mortality, and uterine rupture [1,6].

Most women with BU do not exhibit any symptoms, and the condition is often identified during pregnancy or childbirth when complications occur, or during abdominal surgeries like hysterectomy [3]. In our case, despite having undergone a previous cesarean section, neither the patient nor her family mentioned any uterine defects; after excluding general surgical diagnoses, a diagnostic laparotomy was conducted to identify the confirmed diagnoses.

Uterine rupture is commonly linked to a history of cesarean sections and multiple pregnancies. Additional risk factors include uterine anomalies, certain obstetric procedures, abnormal placentation, malpresentation, curettage, improper use of oxytocin, and long-term corticosteroid therapy [7]. In our case, a bicornuate uterus was identified as a significant risk factor, along with a prior cesarean section.

Uterine rupture manifests as an acute abdominal state that necessitates considering various obstetric, gynecologic and surgical differential diagnoses. Clinical presentation may include: acute abdominal pain, vaginal hemorrhage, and fetal distress and decelerations [8]. Obstetric and gynecologic differential diagnoses vary from ectopic pregnancy to ovarian torsion and ovarian cyst rupture [9]. In our case, the patient arrived at the emergency department with loss of consciousness, tenderness of abdomen, low blood pressure, and rapid heart rate, all indicating potential hypovolemia. A bedside ultrasound subsequently verified the existence of an indefinite massive free fluid in the abdomen.

The recommended management is prompt surgical resection of the ruptured horn. The subsequent procedure depends on the patient's desire of fertility. After counselling the patient about her plans and confirming her consent of termination of reproduction, hysterectomy is recommended, otherwise, the obstetrician needs to repair the uterus [3]. Our approach involved the prompt administration of blood, blood products, and fluid resuscitation to stabilize the mother's hemodynamics. This was followed by a diagnostic laparotomy to excision the ruptured left uterine horn and repair the uterus.

Regarding possible postoperative complications, a contraceptive plan should be discussed with the patient for at least a year after the operation.

As the pregnant suffering from uterus rupture will present with an acute abdominal state which will be challenging for the obstetrician to define the diagnosis, a rapid and prompt management should be administered regarding the patient's desire of fertility.

## 4 Conclusion

A bicornuate uterus (BU) is a rare congenital anomaly that may present with various obstetric complications. Uterine rupture should be considered as a differential diagnosis in each acute abdominal obstetric case at any gestational age. The initial management should include maintaining a stabilized maternal hemodynamic state by replacing blood products and fluids, followed by surgical intervention.

## Consent for publication

Written informed consent was obtained from the patient for publication of this case report and accompanying images. A copy of the written consent is available for review by the Editor-in-Chief of this journal on request.

## Ethics approval and consent to participate

Not required for case reports at our hospital. Single case reports are exempt from ethical approval in our institution.

## Ethics approval and consent to participate

This study is exempt from ethical approval in our institution (Obstetrics and Gynecology University Hospital of Aleppo, Faculty of Medicine, University of Aleppo, Aleppo, Syria) because the content of the single case report does not require ethical approval.

## Submission declaration

The article is not under consideration for publication elsewhere.

## Funding

This research did not receive any specific grant from funding agencies in the public, commercial, or not-for-profit sectors.

## Declaration of competing interest

None.
